# μ-Peroxido-bis­[acetonitrile­bis­(ethyl­enediamine)­cobalt(III)] tetrakis(per­chlorate)

**DOI:** 10.1107/S1600536810047653

**Published:** 2010-11-24

**Authors:** Khrystyna O. Regeta, Iryna Odarich, Svetlana V. Pavlova, Valentina A. Kalibabchuk, Matti Haukka

**Affiliations:** a National Taras Shevchenko University, Department of Chemistry, Volodymyrska Str. 64, 01601 Kiev, Ukraine; b Bohomolets National Medical University, Department of General Chemistry, Shevchenko Blvd 13, 01004 Kiev, Ukraine; c Department of Chemistry, University of Joensuu, PO Box 111, 80101 Joensuu, Finland

## Abstract

The title compound, [Co_2_(O_2_)(CH_3_CN)_2_(C_2_H_8_N_2_)_4_](ClO_4_)_4_, consists of centrosymmetric binuclear cations and perchlorate anions. Two Co^III^ atoms, which have a slightly distorted octa­hedral coordination, are connected through a peroxido bridge; the O—O distance is 1.476 (3) Å. Both acetonitrile ligands are situated in a *trans* position with respect to the O—O bridge. In the crystal, the complex cations are connected by N—H⋯O hydrogen bonds between ethyl­endiamine NH groups and O atoms from the perchlorate anions and peroxide O atoms.

## Related literature

For related structures, see: Shibahara *et al.* (1973 ▶); Dexter *et al.* (1984 ▶); Sliva *et al.* (1997 ▶); Petrusenko *et al.* (1997 ▶); McMullen & Hagen (2002 ▶); Mokhir *et al.* (2002 ▶); Sliva *et al.* (1997 ▶); Wörl *et al.* (2005 ▶). For of applications di­oxy­gen cobalt complexes, see: Busch & Alcock (1994 ▶), Jain & Sain (2003 ▶).
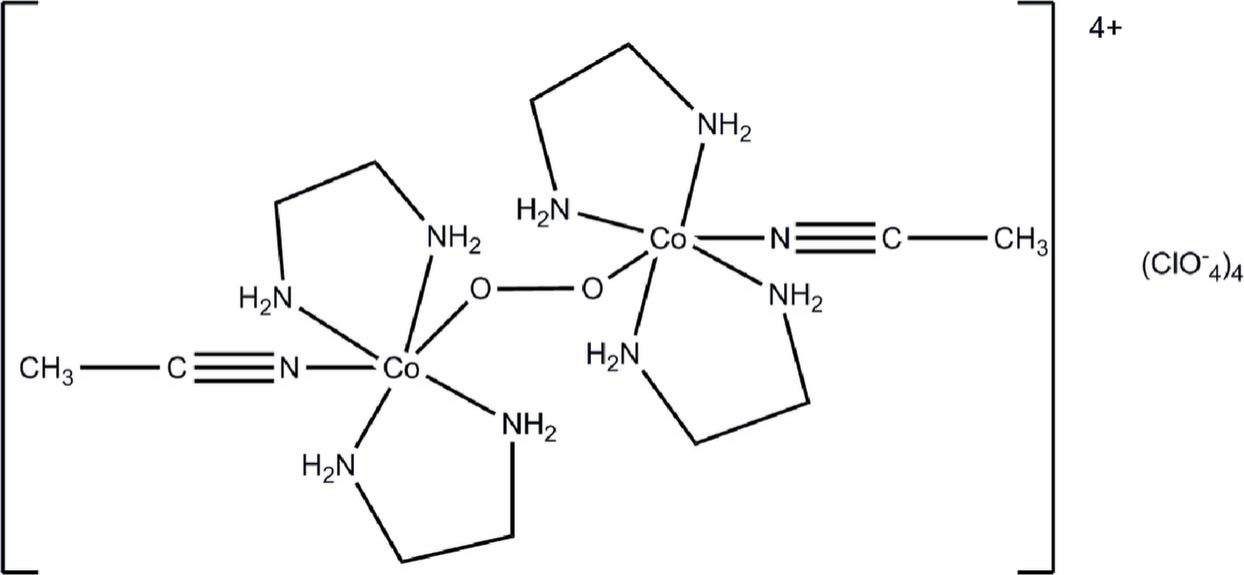


## Experimental

### 

#### Crystal data

[Co_2_(O_2_)(C_2_H_3_N)_2_(C_2_H_8_N_2_)_4_](ClO_4_)_4_
*M* *_r_* = 870.18
Monoclinic, 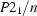

*a* = 11.9747 (7) Å
*b* = 8.3348 (6) Å
*c* = 16.4921 (10) Å
β = 109.702 (5)°
*V* = 1549.66 (17) Å^3^
*Z* = 2
Mo *K*α radiation
μ = 1.51 mm^−1^
*T* = 100 K
0.40 × 0.14 × 0.12 mm

#### Data collection

Nonius KappaCCD diffractometer
Absorption correction: multi-scan (*SADABS*; Bruker, 2004 ▶) *T*_min_ = 0.584, *T*_max_ = 0.838
29196 measured reflections
3550 independent reflections
2868 reflections with *I* > 2σ(*I*)
*R*_int_ = 0.044

#### Refinement

*R*[*F* ^2^ > 2σ(*F* ^2^)] = 0.028
*wR*(*F* ^2^) = 0.067
*S* = 1.04
3550 reflections
209 parameters
H-atom parameters constrained
Δρ_max_ = 0.45 e Å^−3^
Δρ_min_ = −0.38 e Å^−3^

### 

Data collection: *COLLECT* (Bruker, 2004 ▶); cell refinement: *DENZO*/*SCALEPACK* (Otwinowski & Minor, 1997 ▶); data reduction: *DENZO*/*SCALEPACK*; program(s) used to solve structure: *SHELXS97* (Sheldrick, 2008 ▶); program(s) used to refine structure: *SHELXL97* (Sheldrick, 2008 ▶); molecular graphics: *DIAMOND* (Bradenburg, 2006 ▶); software used to prepare material for publication: *SHELXL97*.

## Supplementary Material

Crystal structure: contains datablocks I, global. DOI: 10.1107/S1600536810047653/vm2054sup1.cif

Structure factors: contains datablocks I. DOI: 10.1107/S1600536810047653/vm2054Isup2.hkl

Additional supplementary materials:  crystallographic information; 3D view; checkCIF report

## Figures and Tables

**Table 1:** Selected bond lengths (Å)

Co1—O1	1.8640 (13)
Co1—N5	1.9289 (16)
Co1—N2	1.9382 (17)
Co1—N3	1.9430 (17)
Co1—N4	1.9533 (17)
Co1—N1	1.9565 (17)

**Table 2:** Hydrogen-bond geometry (Å, °)

*D*—H⋯*A*	*D*—H	H⋯*A*	*D*⋯*A*	*D*—H⋯*A*
N1—H1*N*⋯O4^ii^	0.82	2.18	2.989 (2)	168
N1—H1*M*⋯O6	0.95	2.12	2.945 (2)	145
N2—H2*N*⋯O5^iii^	0.87	2.26	3.084 (2)	158
N2—H2*M*⋯O1^i^	0.77	2.31	2.860 (2)	129
N3—H3*N*⋯O8^iv^	0.86	2.29	3.094 (2)	156
N3—H3*M*⋯O1^i^	0.89	2.17	2.735 (2)	120
N3—H3*M*⋯O7^i^	0.89	2.26	3.042 (2)	146
N4—H4*N*⋯O2	0.80	2.23	3.000 (2)	160
N4—H4*M*⋯O9^v^	0.83	2.57	3.266 (2)	142

Symmetry codes: (i) 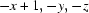; (ii) 
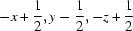
; (iii) 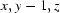; (iv) 
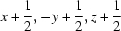
; (v) 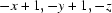.

**Fig. 1.**
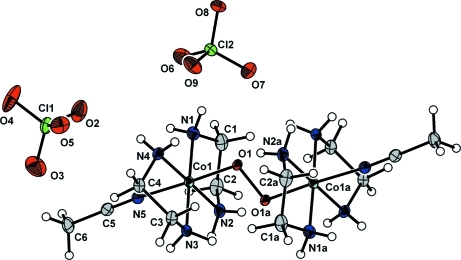
The molecular structure of (I), with 50% probability displacement ellipsoids showing the atom-numbering scheme employed. As molecule is centrosymmetric, two perchlorate groups are omitted. [Symmetry code: (a) 1 - x, -y, -z].

**Fig. 2.**
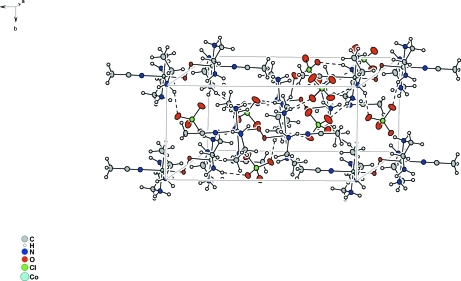
A packing diagram for compound (I). Hydrogen bonds are indicated by dashed lines.

## supplementary crystallographic information

### Comment

Dioxygen complexes have been investigated in order to understand the mechanisms of oxygen metabolism such as O_2_ transport, storage and activation, which are essential events for life. The dioxygen cobalt complexes attract a lot of attention because of their potential use as artificial oxygen carriers (Busch *et al.*, 1994) and industrial oxidation catalysts (Jain *et al.*, 2003), for example: the *p*-xylene oxidation giving terephtalic acid and the adipic acid synthesis from cyclohexane. In many cases, reactions of cobalt (II) complexes with dioxygen proceeds, however, irreversibly resulting in formation of cobalt (III) complexes, and very often the intermediate products of such reactions appear to be binuclear cobalt (III) peroxo species. The title compound (I) was obtained as a result of reaction of cobalt (II) perchlorate in acetonitrile and ethylendiamine. The crystal structure of (I) consists of cationic dicobalt(III) *µ**_2_*-peroxo complexes and perchlorate anions. The molecules are centrosymmetric. The Co (III) ions are six-coordinated, the axial positions are occupied by acetonitrile and peroxo bridge, the ethylendiamine ligands lie in the equatorial plane. The acetonitriles ligands are *trans* with respect to the O—O bridge. The analysis of the bond lengths and angles of (I) indicates that the coordination environment of the cobalt is slightly distorted octahedral. The bond distances Co1—N (CH_3_CN), Co1—O and O—O are 1.9289 (16), 1.8640 (13) and 1.476 (3) Å, respectively. The average of Co—N(en) distances is 1.9478 Å and average of O1—Co—N(en) angles is 89.80°. The bond angle O—Co—N (MeCN) is equal 177.26 (7)°. The C—N, C≡N and C—C bond lengths in the ethylenediamine and acetonitrile ligands are normal and close to the values observed in the related structures (Sliva *et al.*, 1997; Petrusenko *et al.*, 1997; Mokhir *et al.*, 2002; Wörl *et al.*, 2005).

Perchlorate anions do not form direct bonds with cobalt but they are connected to NH groups of ethylendiamine through hydrogen bonds. All of the NH groups of ethylenediamine form hydrogen bonds with either the oxygen atoms of perchlorate anions or the peroxide oxygen atoms (Table 2). A hydrogen-bonding network links the cations and anions into stacks stretching along the *b* axis (Fig. 2).

### Experimental

The title compound was obtained by slow diffusion of ethylendiamine vapours to the air-exposured solution, containing Co(ClO_4_)_2_ (0,1 mmol/*L*) in acetonitrile. The yellow-brown crystals were formed in ten days.

### Refinement

The NH_2_ hydrogen atoms were located from the difference Fourier map but constrained to ride on their parent atom, with *U*_iso_ = 1.5 *U*_eq_(parent atom). Other hydrogen atoms were positioned geometrically and were also constrained to ride on their parent atoms, with C—H = 0.98–0.99 Å, and *U*_iso_ = 1.2–1.5 *U*_eq_(parent atom). The highest peak is located 0.84 Å from atom Co1 and the deepest hole is located 0.57 Å from atom Cl1.

### Crystal data

**Table:**

[Co_2_(O_2_)(C_2_H_3_N)_2_(C_2_H_8_N_2_)_4_](ClO_4_)_4_	*F*(000) = 892
*M**_r_* = 870.18	*D*_x_ = 1.865 Mg m^−^^3^
Monoclinic, *P*2_1_/*n*	Mo *K*α radiation, λ = 0.71073 Å
Hall symbol: -P 2yn	Cell parameters from 6337 reflections
*a* = 11.9747 (7) Å	θ = 1.0–27.5°
*b* = 8.3348 (6) Å	µ = 1.51 mm^−^^1^
*c* = 16.4921 (10) Å	*T* = 100 K
β = 109.702 (5)°	Plate, yellow-brown
*V* = 1549.66 (17) Å^3^	0.40 × 0.14 × 0.12 mm
*Z* = 2	

### Data collection

**Table:**

Nonius KappaCCD diffractometer	3550 independent reflections
Radiation source: fine-focus sealed tube	2868 reflections with *I* > 2σ(*I*)
horizontally mounted graphite crystal	*R*_int_ = 0.044
Detector resolution: 9 pixels mm^-1^	θ_max_ = 27.5°, θ_min_ = 2.6°
φ scans and ω scans with κ offset	*h* = −15→15
Absorption correction: multi-scan (*SADABS*; Sheldrick, 2008)	*k* = −10→10
*T*_min_ = 0.584, *T*_max_ = 0.838	*l* = −21→21
29196 measured reflections	

### Refinement

**Table:**

Refinement on *F*^2^	Primary atom site location: structure-invariant direct methods
Least-squares matrix: full	Secondary atom site location: difference Fourier map
*R*[*F*^2^ > 2σ(*F*^2^)] = 0.028	H-atom parameters constrained
*wR*(*F*^2^) = 0.067	*w* = 1/[σ^2^(*F*_o_^2^) + (0.0258*P*)^2^ + 1.566*P*] where *P* = (*F*_o_^2^ + 2*F*_c_^2^)/3
*S* = 1.04	(Δ/σ)_max_ = 0.001
3550 reflections	Δρ_max_ = 0.45 e Å^−^^3^
209 parameters	Δρ_min_ = −0.38 e Å^−^^3^
0 restraints	

### Special details

**Table:**

Geometry. All e.s.d.'s (except the e.s.d. in the dihedral angle between two l.s. planes) are estimated using the full covariance matrix. The cell e.s.d.'s are taken into account individually in the estimation of e.s.d.'s in distances, angles and torsion angles; correlations between e.s.d.'s in cell parameters are only used when they are defined by crystal symmetry. An approximate (isotropic) treatment of cell e.s.d.'s is used for estimating e.s.d.'s involving l.s. planes.
Refinement. Refinement of *F*^2^ against ALL reflections. The weighted *R*-factor *wR* and goodness of fit *S* are based on *F*^2^, conventional *R*-factors *R* are based on *F*, with *F* set to zero for negative *F*^2^. The threshold expression of *F*^2^ > σ(*F*^2^) is used only for calculating *R*-factors(gt) *etc*. and is not relevant to the choice of reflections for refinement. *R*-factors based on *F*^2^ are statistically about twice as large as those based on *F*, and *R*- factors based on ALL data will be even larger.

### Fractional atomic coordinates and isotropic or equivalent isotropic displacement parameters (Å^2^)

**Table:**

	*x*	*y*	*z*	*U*_iso_*/*U*_eq_	
Co1	0.44371 (2)	0.03919 (3)	0.114097 (16)	0.01180 (8)	
Cl1	0.43255 (5)	0.54025 (6)	0.29786 (3)	0.02212 (12)	
Cl2	0.26746 (4)	0.44096 (6)	−0.07578 (3)	0.01802 (11)	
O1	0.45899 (12)	0.06143 (16)	0.00586 (8)	0.0144 (3)	
O2	0.34823 (15)	0.4558 (2)	0.22821 (13)	0.0433 (5)	
O3	0.49202 (19)	0.4287 (2)	0.36324 (12)	0.0454 (5)	
O4	0.37142 (19)	0.6573 (2)	0.33071 (14)	0.0467 (5)	
O5	0.51665 (15)	0.6197 (2)	0.26689 (11)	0.0317 (4)	
O6	0.21587 (15)	0.3920 (2)	−0.01269 (10)	0.0301 (4)	
O7	0.29139 (18)	0.3044 (2)	−0.11896 (12)	0.0459 (5)	
O8	0.18693 (13)	0.54567 (18)	−0.13697 (9)	0.0207 (3)	
O9	0.37603 (14)	0.5250 (2)	−0.03261 (10)	0.0304 (4)	
N1	0.27241 (15)	0.0668 (2)	0.05928 (11)	0.0178 (4)	
H1N	0.2429	0.0940	0.0954	0.027*	
H1M	0.2599	0.1481	0.0168	0.027*	
N2	0.41301 (15)	−0.1890 (2)	0.09797 (11)	0.0173 (4)	
H2N	0.4591	−0.2454	0.1404	0.026*	
H2M	0.4320	−0.2103	0.0591	0.026*	
N3	0.61375 (15)	0.0074 (2)	0.16628 (11)	0.0149 (3)	
H3N	0.6268	−0.0363	0.2157	0.022*	
H3M	0.6353	−0.0600	0.1320	0.022*	
N4	0.47635 (15)	0.2686 (2)	0.13121 (10)	0.0154 (4)	
H4N	0.4275	0.3112	0.1473	0.023*	
H4M	0.4831	0.3058	0.0863	0.023*	
N5	0.42619 (15)	0.0270 (2)	0.22599 (11)	0.0153 (3)	
C1	0.21715 (19)	−0.0890 (3)	0.02411 (14)	0.0223 (5)	
H1A	0.2200	−0.1055	−0.0346	0.027*	
H1B	0.1332	−0.0910	0.0211	0.027*	
C2	0.2860 (2)	−0.2181 (3)	0.08353 (14)	0.0221 (5)	
H2A	0.2707	−0.2142	0.1389	0.026*	
H2B	0.2623	−0.3250	0.0573	0.026*	
C3	0.67342 (18)	0.1652 (2)	0.17222 (13)	0.0175 (4)	
H3A	0.6826	0.1928	0.1164	0.021*	
H3B	0.7530	0.1620	0.2170	0.021*	
C4	0.59672 (18)	0.2872 (2)	0.19526 (13)	0.0172 (4)	
H4A	0.5955	0.2677	0.2542	0.021*	
H4B	0.6271	0.3969	0.1927	0.021*	
C5	0.42665 (17)	0.0207 (2)	0.29474 (13)	0.0160 (4)	
C6	0.4315 (2)	0.0148 (3)	0.38351 (13)	0.0220 (5)	
H6A	0.4893	−0.0663	0.4146	0.033*	
H6B	0.3531	−0.0133	0.3857	0.033*	
H6C	0.4553	0.1199	0.4104	0.033*	

### Atomic displacement parameters (Å^2^)

**Table:**

	*U*^11^	*U*^22^	*U*^33^	*U*^12^	*U*^13^	*U*^23^
Co1	0.01439 (14)	0.01312 (14)	0.00968 (13)	0.00057 (11)	0.00640 (10)	0.00060 (10)
Cl1	0.0281 (3)	0.0222 (3)	0.0219 (3)	−0.0016 (2)	0.0162 (2)	−0.0029 (2)
Cl2	0.0178 (2)	0.0204 (2)	0.0144 (2)	0.00197 (19)	0.00348 (19)	0.00035 (19)
O1	0.0189 (7)	0.0159 (7)	0.0114 (6)	0.0061 (6)	0.0088 (6)	0.0020 (5)
O2	0.0229 (9)	0.0592 (13)	0.0456 (11)	−0.0001 (9)	0.0090 (8)	−0.0269 (10)
O3	0.0617 (14)	0.0395 (11)	0.0338 (10)	−0.0038 (10)	0.0146 (10)	0.0120 (8)
O4	0.0672 (14)	0.0256 (9)	0.0752 (14)	−0.0083 (9)	0.0606 (12)	−0.0138 (9)
O5	0.0295 (9)	0.0359 (10)	0.0388 (10)	0.0040 (8)	0.0234 (8)	0.0101 (8)
O6	0.0323 (9)	0.0324 (9)	0.0278 (9)	−0.0016 (7)	0.0130 (7)	0.0113 (7)
O7	0.0592 (13)	0.0373 (10)	0.0327 (10)	0.0242 (10)	0.0044 (9)	−0.0101 (8)
O8	0.0179 (7)	0.0237 (8)	0.0187 (7)	0.0035 (6)	0.0036 (6)	0.0061 (6)
O9	0.0185 (8)	0.0451 (10)	0.0232 (8)	−0.0069 (7)	0.0014 (7)	0.0031 (8)
N1	0.0173 (9)	0.0234 (9)	0.0152 (8)	0.0012 (7)	0.0087 (7)	0.0020 (7)
N2	0.0225 (9)	0.0181 (9)	0.0143 (8)	0.0000 (7)	0.0102 (7)	0.0000 (7)
N3	0.0176 (9)	0.0173 (8)	0.0108 (8)	0.0028 (7)	0.0059 (7)	0.0011 (6)
N4	0.0174 (9)	0.0162 (8)	0.0154 (8)	0.0027 (7)	0.0090 (7)	−0.0001 (7)
N5	0.0156 (8)	0.0165 (8)	0.0152 (9)	0.0008 (7)	0.0072 (7)	0.0011 (7)
C1	0.0189 (11)	0.0298 (12)	0.0207 (11)	−0.0058 (9)	0.0099 (9)	−0.0057 (9)
C2	0.0267 (12)	0.0206 (11)	0.0244 (11)	−0.0080 (9)	0.0157 (10)	−0.0038 (9)
C3	0.0169 (10)	0.0200 (10)	0.0169 (10)	−0.0024 (8)	0.0073 (8)	−0.0039 (8)
C4	0.0190 (10)	0.0180 (10)	0.0155 (10)	−0.0012 (8)	0.0070 (8)	−0.0028 (8)
C5	0.0146 (10)	0.0162 (10)	0.0183 (10)	−0.0009 (8)	0.0071 (8)	−0.0009 (8)
C6	0.0234 (11)	0.0306 (12)	0.0153 (10)	−0.0010 (9)	0.0107 (9)	−0.0012 (9)

### Geometric parameters (Å, °)

**Table:**

Co1—O1	1.8640 (13)	N3—C3	1.484 (3)
Co1—N5	1.9289 (16)	N3—H3N	0.8574
Co1—N2	1.9382 (17)	N3—H3M	0.8943
Co1—N3	1.9430 (17)	N4—C4	1.480 (3)
Co1—N4	1.9533 (17)	N4—H4N	0.8014
Co1—N1	1.9565 (17)	N4—H4M	0.8322
Cl1—O3	1.4198 (19)	N5—C5	1.133 (3)
Cl1—O4	1.4313 (17)	C1—C2	1.501 (3)
Cl1—O2	1.4328 (18)	C1—H1A	0.9900
Cl1—O5	1.4353 (16)	C1—H1B	0.9900
Cl2—O7	1.4219 (18)	C2—H2A	0.9900
Cl2—O8	1.4331 (15)	C2—H2B	0.9900
Cl2—O9	1.4364 (16)	C3—C4	1.502 (3)
Cl2—O6	1.4366 (16)	C3—H3A	0.9900
O1—O1^i^	1.476 (3)	C3—H3B	0.9900
N1—C1	1.484 (3)	C4—H4A	0.9900
N1—H1N	0.8204	C4—H4B	0.9900
N1—H1M	0.9493	C5—C6	1.446 (3)
N2—C2	1.478 (3)	C6—H6A	0.9800
N2—H2N	0.8691	C6—H6B	0.9800
N2—H2M	0.7698	C6—H6C	0.9800
			
O1—Co1—N5	177.26 (7)	Co1—N3—H3N	107.9
O1—Co1—N2	92.38 (6)	C3—N3—H3M	111.3
N5—Co1—N2	90.16 (7)	Co1—N3—H3M	106.7
O1—Co1—N3	90.64 (6)	H3N—N3—H3M	109.5
N5—Co1—N3	90.28 (7)	C4—N4—Co1	107.74 (12)
N2—Co1—N3	92.81 (7)	C4—N4—H4N	111.2
O1—Co1—N4	87.80 (6)	Co1—N4—H4N	110.4
N5—Co1—N4	89.68 (7)	C4—N4—H4M	103.5
N2—Co1—N4	179.39 (8)	Co1—N4—H4M	108.1
N3—Co1—N4	86.60 (7)	H4N—N4—H4M	115.5
O1—Co1—N1	88.39 (7)	C5—N5—Co1	173.85 (17)
N5—Co1—N1	90.75 (7)	N1—C1—C2	107.31 (17)
N2—Co1—N1	86.05 (7)	N1—C1—H1A	110.3
N3—Co1—N1	178.47 (7)	C2—C1—H1A	110.3
N4—Co1—N1	94.54 (7)	N1—C1—H1B	110.3
O3—Cl1—O4	110.39 (13)	C2—C1—H1B	110.3
O3—Cl1—O2	108.94 (13)	H1A—C1—H1B	108.5
O4—Cl1—O2	109.05 (12)	N2—C2—C1	107.26 (17)
O3—Cl1—O5	109.85 (11)	N2—C2—H2A	110.3
O4—Cl1—O5	109.27 (10)	C1—C2—H2A	110.3
O2—Cl1—O5	109.31 (11)	N2—C2—H2B	110.3
O7—Cl2—O8	109.63 (10)	C1—C2—H2B	110.3
O7—Cl2—O9	109.68 (12)	H2A—C2—H2B	108.5
O8—Cl2—O9	109.51 (10)	N3—C3—C4	107.14 (16)
O7—Cl2—O6	110.11 (12)	N3—C3—H3A	110.3
O8—Cl2—O6	109.36 (9)	C4—C3—H3A	110.3
O9—Cl2—O6	108.53 (10)	N3—C3—H3B	110.3
O1^i^—O1—Co1	109.93 (12)	C4—C3—H3B	110.3
C1—N1—Co1	109.73 (13)	H3A—C3—H3B	108.5
C1—N1—H1N	106.3	N4—C4—C3	106.26 (16)
Co1—N1—H1N	109.7	N4—C4—H4A	110.5
C1—N1—H1M	113.4	C3—C4—H4A	110.5
Co1—N1—H1M	107.7	N4—C4—H4B	110.5
H1N—N1—H1M	109.9	C3—C4—H4B	110.5
C2—N2—Co1	108.69 (13)	H4A—C4—H4B	108.7
C2—N2—H2N	112.4	N5—C5—C6	178.0 (2)
Co1—N2—H2N	112.4	C5—C6—H6A	109.5
C2—N2—H2M	113.9	C5—C6—H6B	109.5
Co1—N2—H2M	104.2	H6A—C6—H6B	109.5
H2N—N2—H2M	105.0	C5—C6—H6C	109.5
C3—N3—Co1	108.53 (12)	H6A—C6—H6C	109.5
C3—N3—H3N	112.6	H6B—C6—H6C	109.5

Symmetry codes: (i) −*x*+1, −*y*, −*z*.

### Hydrogen-bond geometry (Å, °)

**Table:**

*D*—H···*A*	*D*—H	H···*A*	*D*···*A*	*D*—H···*A*
N1—H1N···O4^ii^	0.82	2.18	2.989 (2)	168
N1—H1M···O6	0.95	2.12	2.945 (2)	145
N2—H2N···O5^iii^	0.87	2.26	3.084 (2)	158
N2—H2M···O1^i^	0.77	2.31	2.860 (2)	129
N3—H3N···O8^iv^	0.86	2.29	3.094 (2)	156
N3—H3M···O1^i^	0.89	2.17	2.735 (2)	120
N3—H3M···O7^i^	0.89	2.26	3.042 (2)	146
N4—H4N···O2	0.80	2.23	3.000 (2)	160
N4—H4M···O9^v^	0.83	2.57	3.266 (2)	142

Symmetry codes: (ii) −*x*+1/2, *y*−1/2, −*z*+1/2; (iii) *x*, *y*−1, *z*; (i) −*x*+1, −*y*, −*z*; (iv) *x*+1/2, −*y*+1/2, *z*+1/2; (v) −*x*+1, −*y*+1, −*z*.
